# First record and redescription of the terrestrial isopod *Hemilepistoides messerianus* Borutzky, 1945 (Isopoda, Oniscidea) from Iran

**DOI:** 10.3897/zookeys.515.9179

**Published:** 2015-07-30

**Authors:** Ghasem M. Kashani

**Affiliations:** 1 Department of Biology, Faculty of Science, University of Zanjan, Zanjan, Iran

**Keywords:** Oniscidea, *Hemilepistoides messerianus*, redescription, Turkmenistan, Iran

## Abstract

In the present study, *Hemilepistoides messerianus* Borutzky, 1945 is reported from Iran for the first time. This species is redescribed and diagnostic characters of both males and females are illustrated. This species is characterized by the tuberculation of all parts of the dorsal surface of the body and the male pleopod endopodite I with a triangular lobe at apex. A map with the distribution of species is presented.

## Introduction

Among a dozen species of terrestrial isopods from Turkmenia [now Turkmenistan], Borutzky (1945) established the new genus and species *Hemilepistoides messerianus* for two female specimens collected from Ashgabat region. No further study on this taxon has been published since. The type material was deposited in the Zoological Museum of Moscow State University (ZMMU) (Borutzky 1972). Re-examination of type material of terrestrial isopods in ZMMU revealed that the type specimens of *Hemilepistoides messerianus* are presumably lost.

In a survey on terrestrial isopods of northern Iran, many specimens belonging to the genus *Hemilepistoides* were found. Comparison of female specimens collected from Iran with description and illustrations presented by Borutzky (1945) for *Hemilepistoides messerianus* revealed no marked difference between them, convincing that they belong to the same species.

The aim of the present study is to redescribe *Hemilepistoides messerianus* on the basis of both female and male specimens from Iran.

## Material and methods

All material was collected by the author in northern Iran. The specimens were collected by hand and preserved in 96% ethanol. The isopods were dissected and body parts were mounted in micropreparations using Euparal (Carl Roth, Karlsruhe). Drawings were made using a drawing tube fitted on a Nikon Y-IDT compound microscope. Micrographs were taken using a Hitachi S-2460N SEM.

**Figure 1.**
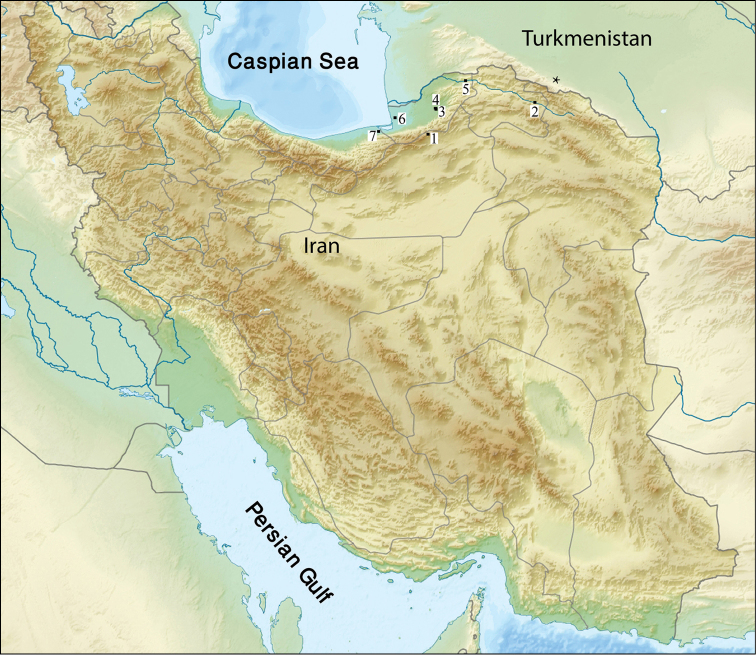
Sampling localities of *Hemilepistoides messerianus* in Iran and position of the type locality (asterisk) in Turkmenistan. The numbers refer to localities listed in the material examined.

Some material of the present study was deposited in the Zoological Museum, University of Tehran (ZUTC) and Iranian Research Institute of Plant Protection (IRIPP), and the others were kept in the personal collection of the author (PCGMK). A map with sampling localities for *Hemilepistoides messerianus* in Iran along with the type locality is presented (Fig. 1).

## Taxonomy

### Order Isopoda Latreille, 1817 Suborder Oniscidea Latreille, 1802 Family Agnaridae Schmidt, 2003

#### Genus Hemilepistoides

Taxon classificationAnimaliaIsopodaAgnaridae

Borutzky, 1945

##### Type species.

*Hemilepistoides messerianus* Borutzky, 1945

##### Diagnosis.

Body narrow and elongated, dorsal parts bearing rounded tubercles. Head with developed lateral lobes; frons with a distinct incision in the middle; no supra-antennal line. Antenna with flagellum of two articles, proximal segment longer than distal one. Pereon epimera I with rounded posterolateral margin. Pleotelson triangular with rounded apex and slightly concave sides. Male pereopods I-II with brushes of setae on sternal margin of merus and carpus; pereopod VII with sinuate sternal margin. Pleopod exopodites I-V with monospiracular covered lungs. Runner type according to the eco-morphological classification proposed by Schmalfuss (1984).

##### Remarks.

Among the members of the family Agnaridae, the genus *Hemilepistoides* is similar to the members of the subgenus Hemilepistus (Desertellio), from which it differs in possessing tubercles also on all posterior parts of the body. The genus *Hemilepistoides* is a monotypical taxon with *Hemilepistoides messerianus* distributed in southern Turkmenistan and northern Iran.

#### Hemilepistoides messerianus

Taxon classificationAnimaliaIsopodaAgnaridae

Borutzky, 1945

##### Material examined.

Semnan, [1] Shahrood, Kalate-Khij, 36°40.1'N, 55°18.7'E, 6 May 2008, two females (ZUTC 5327); the same data as before, two males and two females (PCGMK 1178); [2] Khorasan Shomali, 5 km W of Shirvan, 37°25.1'N, 57°52.7'E, 7 May 2008, one male and one female (ZUTC 5328); same data, two males and nine females (PCGMK 1182); [3] Golestan, S of Gonbade-Kavoos, 37°13.3'N, 55°09.8'E, 10 September 2008, two males and one female (PCGMK 1308); [4] N of Gonbade-Kavoos, 37°16.0'N, 55°10.0'E, 10 September 2008, two males and one females (ZUTC 5329); same data, ten males and ten females (PCGMK 1309); [5] 7 km E of Maraveh-Tappeh, 37°54.6'N, 56°02.2'E, 2 August 2014, one female (IRIPP Iso-1052); [6] 10 km N of Gomishan, 37°54.6'N, 56°02.2'E, 4 August 2014, one male, two females (IRIPP Iso-1053); [7] Mazandaran, 3 km E of Behshahr, 36°22.5'N, 53°38.8'E, 4 August 2014, one female (IRIPP Iso-1054).

##### Diagnosis.

Cephalothorax with rounded lateral lobes, frons with incision in the middle; dorsal parts of the body bearing rounded tubercles. Male pleopod endopdite I straight, with a leaf-like lobe at apex.

##### Redescription.

Maximum length of both male and female: 15 mm. Body elongated, three times as long as wide. Color: cephalothorax and pleon dark; pereon, pale brown with a median longitudinal dark band or thoroughly dark with pale epimera.

**Figure 2.**
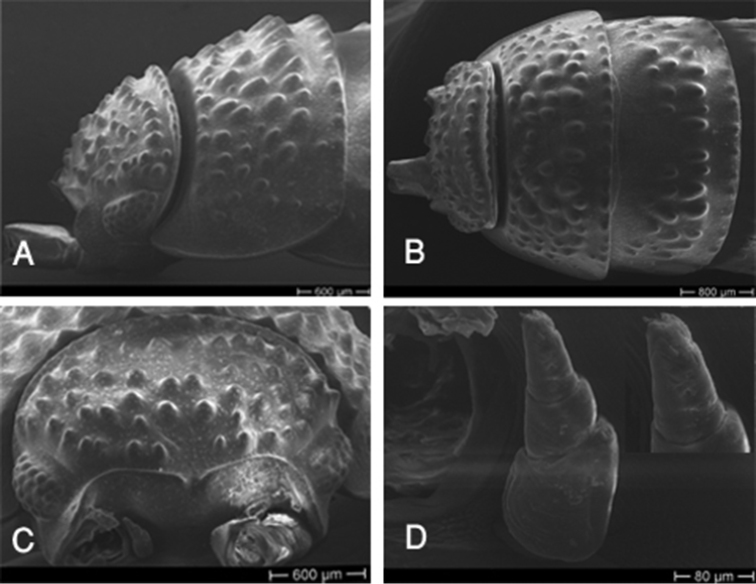
*Hemilepistoides messerianus*, female, from [2]. **A** Head and first pereonite, lateral view **B** head and first pereonite, dorsal view **C** head, frontal view **D** antennule and enlarged distal article.

**Figure 3.**
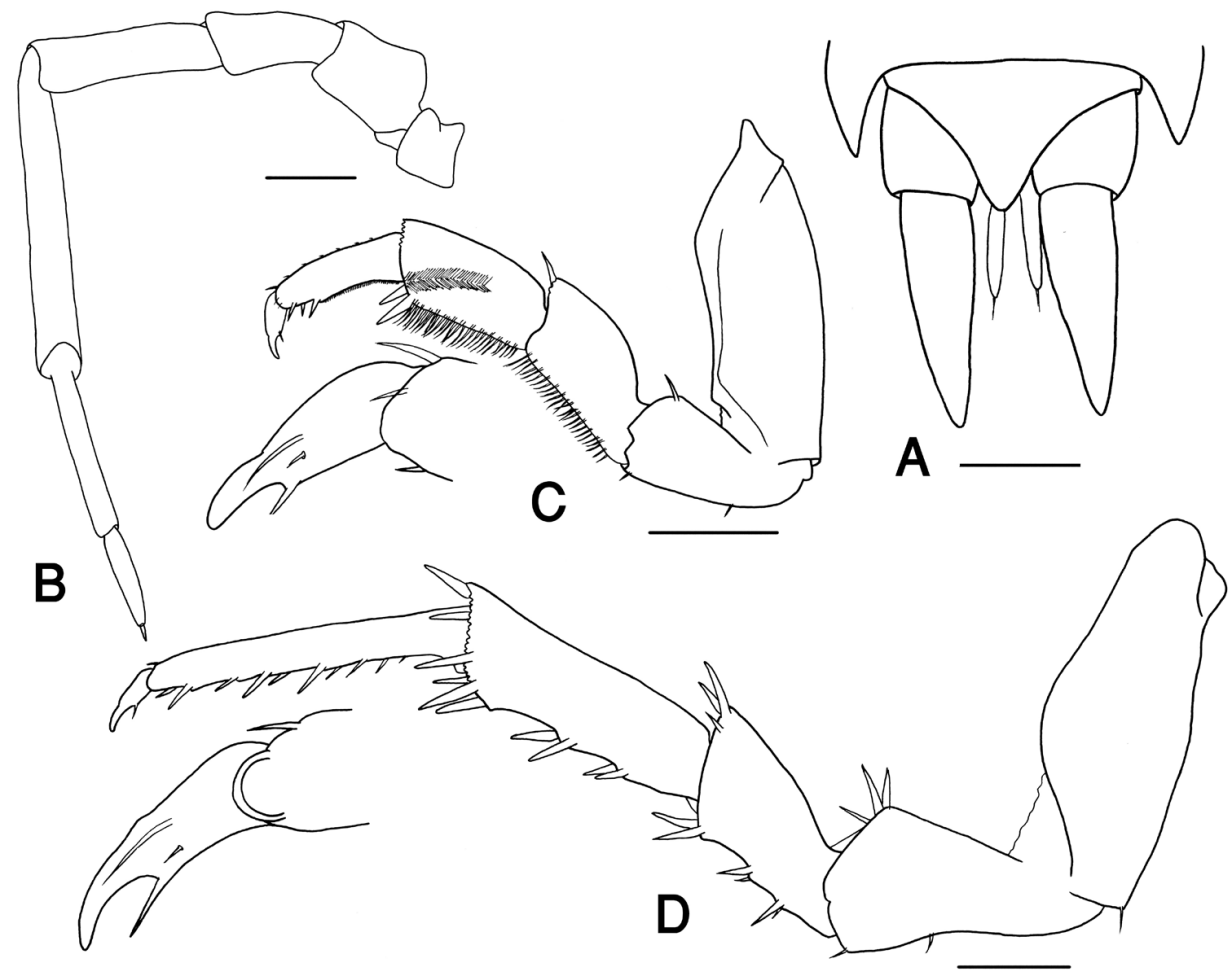
*Hemilepistoides messerianus*, male, from [2]. **A** Telson and uropods **B** antenna **C** pereopod 1 **D** pereopod 7. scales: 0.5 mm.

Cephalothorax with developed rounded lateral lobes, vertex with several rounded tubercles of almost the same size; frontal line sinuous in frontal view, with a distinct incision in the middle; no supraantennal line (Fig. 2C); eyes with 20–24 ommatidia. Antenna long, reaching posterior margin of the second pereon-tergite; flagellum slightly shorter than fifth article of peduncle, with two articles, first article about twice as long as second (Fig. 3B). Antennule of three articles with a tuft of short aesthetascs at apex (Fig. 2D). Pereon-tergites with rounded tubercles, arranged in several rows on the first tergite, median tubercles larger than lateral ones, decreasing in number on posterior tergites. Pereon-tergite I with rounded posterolateral margins (Fig. 2A–B).

**Figure 4.**
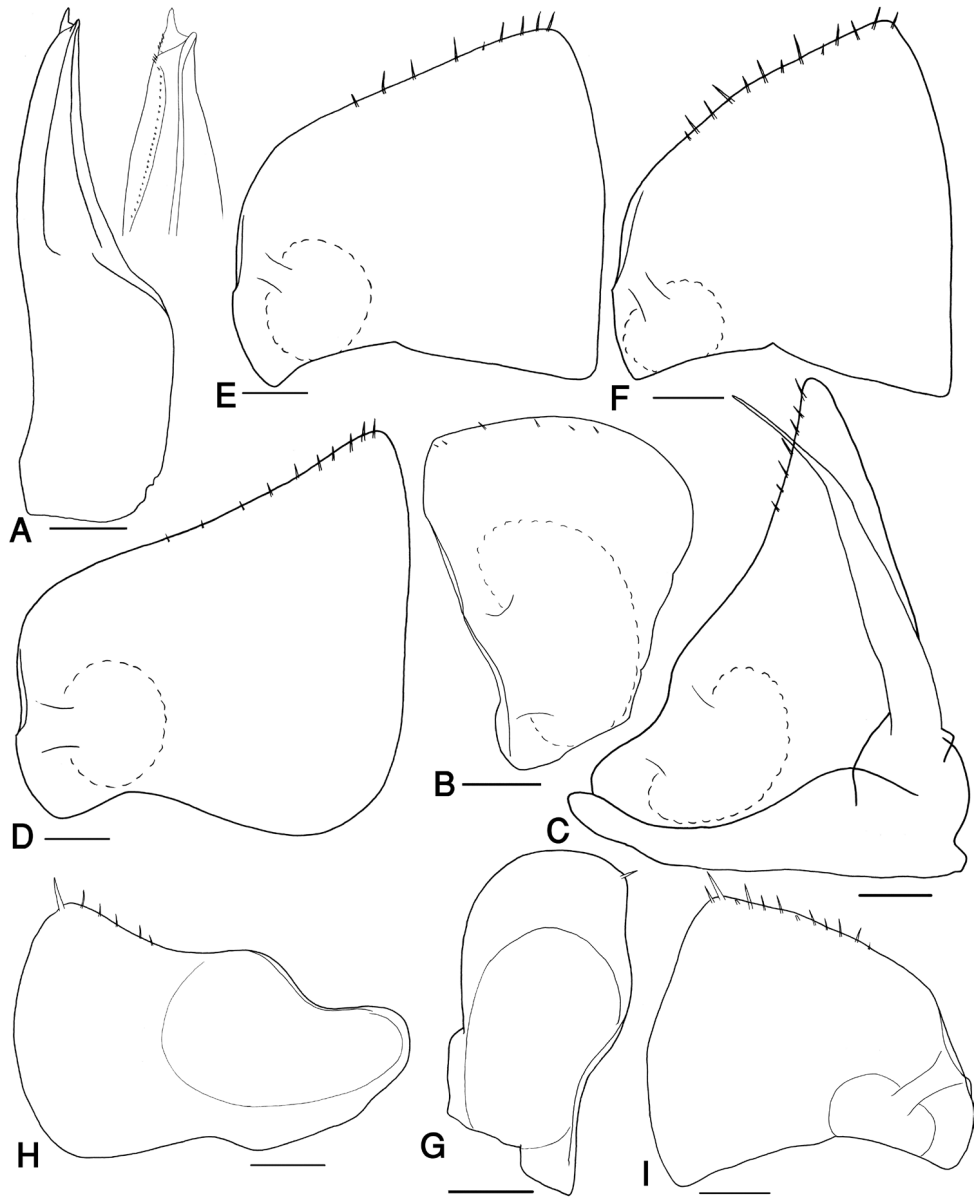
*Hemilepistoides messerianus*, **A–F** male from [3] **G–I** female from [6]. **A** pleopod endopodite I **B** pleopod exopodite I **C** pleopod II **D** pleopod exopodite III **E** pleopod exopodite IV **F** pleopod exopodite V **G** pleopod exopodite I **H** pleopod exopodite II **I** pleopod exopodite V. Scales: 0.2 mm.

Pleon slightly narrower than pereon, each pleon-tergite with a row of faint tubercles on the posterior margin. Pleotelson triangular, with slightly concave sides and rounded apex. Uropod exopodites conical, about 1.5 times as long as pleotelson (Fig. 3A). Pleopod exopodites I-V with monospiracular covered lungs (Fig. 4B–I).

Male: Pereopod I merus and carpus with brushes of setae on sternal margin; propodus narrow and long, proximal part of sternal margin with dense small scales, distal part bearing strong setae (Fig. 3C). Pereopod II merus and carpus with brushes of setae on ventral margin. Pereopod VII ischium with sinuate sternal margin; merus and carpus equipped with strong setae; propodus narrow and long (Fig. 3D). Pleopod endopodite I straight, apex with a triangular lobe, equipped with a row of small setae on inner margin (Fig. 4A). Pleopod exopodite I with a short rounded hind lobe; inner margin with a row of small setae (Fig. 4B). Pleopod exopodite II triangular, with a row of setae on outer margin; endopodite slightly longer than exopodite (Fig. 4C). Pleopod exopodites III-V as in Fig. 4D–F.

Female: Pereopod I merus and carpus without brushes of setae on sternal margin; pereopod VII ischium with straight sternal margin. Pleopod exopodite I with a rounded hind lobe bearing a single spine seta at apex (Fig. 4G). Pleopod exopodite II with two rounded lobes on posterior margin, inner lobe longer than the outer one; a row of setae on posterior margin of inner lobe (Fig. 4H). Pleopod exopodite V as in Fig. 4I, very similar to that of males.

##### Remarks.

Male characteristics are vital for species identification in most terrestrial isopods (Schmidt 2002). *Hemilepistoides messerianus* was described on the basis of female specimens; therefore the identification of the specimens found in Iran with this species might be problematic. Since this species is relatively broadly distributed in northern Iran, the type locality (Ashqabat, Turkmenistan) is not very far from the geographical range of the species in Iran (Fig. 1), and the female characteristics of the Iranian specimens are similar to those of type material described and illustrated by Borutzky (1945), it seems reasonable that they belong to the same species.

This species is distinguished by the shape of the male pleopod endopodite I, with apex bearing a triangular lobe.

##### Distribution.

Southern Turkmenistan; northern Iran.

## Supplementary Material

XML Treatment for Hemilepistoides

XML Treatment for Hemilepistoides messerianus
